# Developing an ecological approach to physical activity promotion in adults with Cystic fibrosis

**DOI:** 10.1371/journal.pone.0272355

**Published:** 2022-08-01

**Authors:** James Shelley, Ellen A. Dawson, Lynne M. Boddy, Claire E. Stewart, Freddy Frost, Dilip Nazareth, Martin J. Walshaw, Zoe R. Knowles

**Affiliations:** 1 Research Institute for Sport and Exercise Sciences, Liverpool John Moores University, Liverpool, United Kingdom; 2 Liverpool Heart and Chest Hospital, Department of Respiratory Medicine, Liverpool, United Kingdom; 3 Institute of Infection and Global health, University of Liverpool, Liverpool, United Kingdom; Universite de Toulon, FRANCE

## Abstract

**Background:**

There are few examples of interventions designed to promote physical activity (PA) in adults with Cystic fibrosis (CF). Increasing levels of habitual PA may be more feasible and result in greater compliance than conventional exercise training inventions which give little or no attention to long-term PA behaviour. Despite this there is limited research exploring perceptions of PA among adults with CF. The study aimed to understand the ecological correlates of PA in adults with CF and to involve individuals with CF, their families (where applicable) and clinicians in a formative process to inform the development of an ecological approach to PA promotion in this population.

**Methods:**

An iterative approach was utilised, whereby findings from earlier phases of the research informed subsequent phases. Semi-structured interviews were conducted to explore patients’ perceptions of PA, devised using the PRECEDE component of the PRECEDE-PROCEED model. Followed by, focus groups to discuss the perceived barriers, facilitators and opportunities for PA participation and how this information could inform the development and delivery of a PA intervention. Separate focus groups were conducted with individuals with CF (n = 11) and their families and CF MDT members. Thematic analysis was used to construct themes.

**Results:**

Physical and mental wellbeing manifested as both barriers and facilitators of PA. CF is characterised by a progressive decline in physical function, which presents as a number of challenging symptoms and set-backs for an individual with CF. PA represents an opportunity for participants to slow the rate of this decline and manage the symptoms associated with the condition. Enjoyment was an important facilitator of PA. Exercise professionals and family reinforce PA behaviour, particularly during adolescence.

**Conclusions:**

PA promotion should form part of routine CF care with additional exercise professional support during adolescence.

## 1. Introduction

Cystic fibrosis (CF) is a genetically inherited condition, predominantly characterised by a variety of life-long systemic complications. Respiratory symptoms are most prevalent, particularly coughing and shortness of breath and as such can lead to fatigue and sleep disturbances [1]. Other complications include gastrointestinal, metabolic and muscoskeletal dysfunction [2] presenting patients with a significant symptom and treatment burden [1]. The promotion of physical activity (PA), which may include structured exercise is recommended as part of routine CF care [3,4]. Despite this there are few examples of interventions designed to promote PA in this population [5]. A large proportion of research in the area has investigated the delivery of exercise training interventions, which give little or no attention to behaviour change theory or long-term maintenance [6]. Additionally, evidence supporting a positive impact of exercise training interventions on clinical outcomes remains equivocal [6]. There is evidence to suggest that higher levels of PA are associated with positive effects on lung function [7] aerobic capacity [8], hospitalisation frequency [9] and mortality [10] in individuals with CF. Translating this evidence into clinical practice has had limited success, though it has previously been proposed that increasing levels of habitual PA may be more feasible and result in greater compliance than conventional exercise training inventions [5]. Despite this, there are few examples of research exploring perceptions of PA among adults with CF and despite many of the barriers and facilitators identified being similar to those already identified in children such as fluctuating health and competing interests, some were specific to adult populations and typically associated with advanced disease severity which may warrant further investigation [11,12]. As with a recent systematic review of qualitative studies exploring correlates of PA among children with CF, the current research seeks to utilise a sociological model to understand the multiple layers of influence on PA behaviour, which is yet to done in adults with CF [13].

The systematic development of interventions, based on the best evidence available and appropriate theory is recommended as best practice. The Medical Research Council (MRC) recommends a phased approach to intervention development with attention given to evaluation throughout [14,15]. Interventions to promote behaviour change, such as increasing PA, should utilise an appropriate conceptual health promotion model and prioritise key factors of the target group [16]. On an individual and population level, PA is a complex and multi-faceted behaviour and as such, socioecological models are a means by which to understand the interacting variables or correlates which have the potential to direct, influence and facilitate behaviour. One such model is the PRECEDE-PROCEED (P-P) model [17]. which is consistent with a socio-ecological model of health promotion and is designed to provide a framework to explain health behaviours and environments to inform the design and evaluation of interventions [17]. Involving participants and their families in a formative process to explore attitudes, norms and perceptions and in the development process is central to a phased approach to complex intervention design [15]. The current paper describes a formative process to explore the perceptions of PA among adults with CF, their families and clinicians. The research adopts a constructivist approach in interpreting the qualitative interview/focus group data and employs reflexive thematic analysis utilising deductive and inductive coding [18] to construct themes centred around the P-P model [17]. Stakeholder (patients, practitioners and policy makers) involvement in the planning, development and implementation of interventions is termed ‘participatory research’ and can provide insights into the ‘real world’ applications of interventions [19]. Therefore, to translate the evidence supporting the beneficial effects of PA in adults with CF into clinical practice it is necessary to involve patients, their families and clinicians in a process to understand the correlates of PA behaviour and to inform the promotion of PA in adults with CF.

## 2. Aim

The aims of the current research were to understand the ecological correlates of PA in adults with CF and to involve individuals with CF, their families (where applicable) and clinicians in a formative process to inform the development of an ecological approach to PA promotion in this population.

## 3. Methods

### 3.1 Participants

Ethical approval was granted by the London–Queen Square Research Ethics Committee and NHS Health Research Authority (19/LO/0305). Participants with CF were recruited from outpatient CF clinics at the regional stand-alone adult CF Centre at Liverpool Heart and Chest NHS Foundation Trust (LHCH). There were no specific inclusion/exclusion criteria applied, beyond being an adult with a confirmed diagnosis of CF (sweat chloride > 60 mmol·L-1 > 100 mg sweat, where possible, diagnostic genotyping) Additionally, members of the CF multi-disciplinary team (MDT) responsible for participants care at LHCH were recruited to participate in phase 2. Recruitment aimed to include representatives from each discipline within the MDT (i.e. Consultant, Physiotherapist, Physiologist, Dietitian and Nurse) and informed written consent was obtained prior to data collection.

### 3.2 Data collection

**Fig 1:**
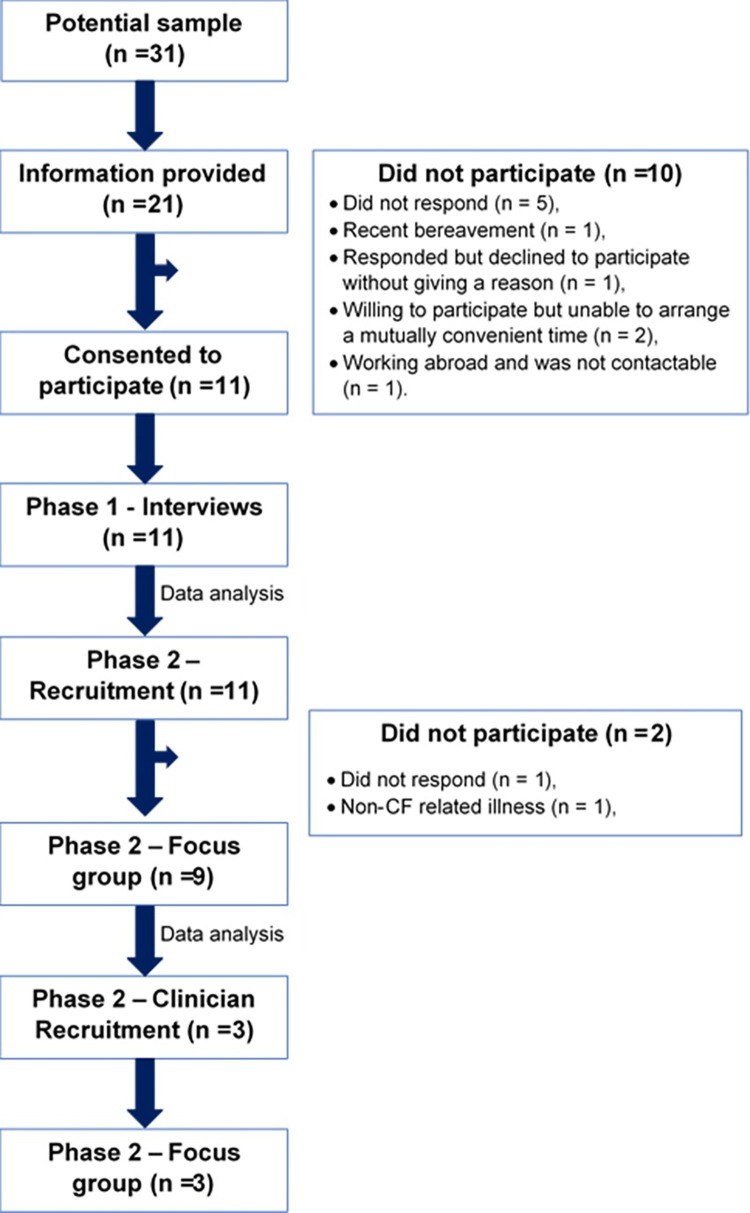
Illustrating the iterative process from participant screening and recruitment to data collection and analysis.

An iterative approach was utilised, whereby findings from earlier phases of the research informed subsequent phases (Fig 1). The data collection and analysis were conducted by a researcher outside of the usual clinical care team but with prior knowledge and experience working with this population in a clinical setting. The semi-structured interview guides and focus group schedules included open-ended questions structured to facilitate open discussion centred on the P-P model. The interview guides were refined through discussion with a second researcher, the final versions and are available as supporting information(S1–S3 Files).

#### 3.2.1. Phase 1

**Fig 2:**
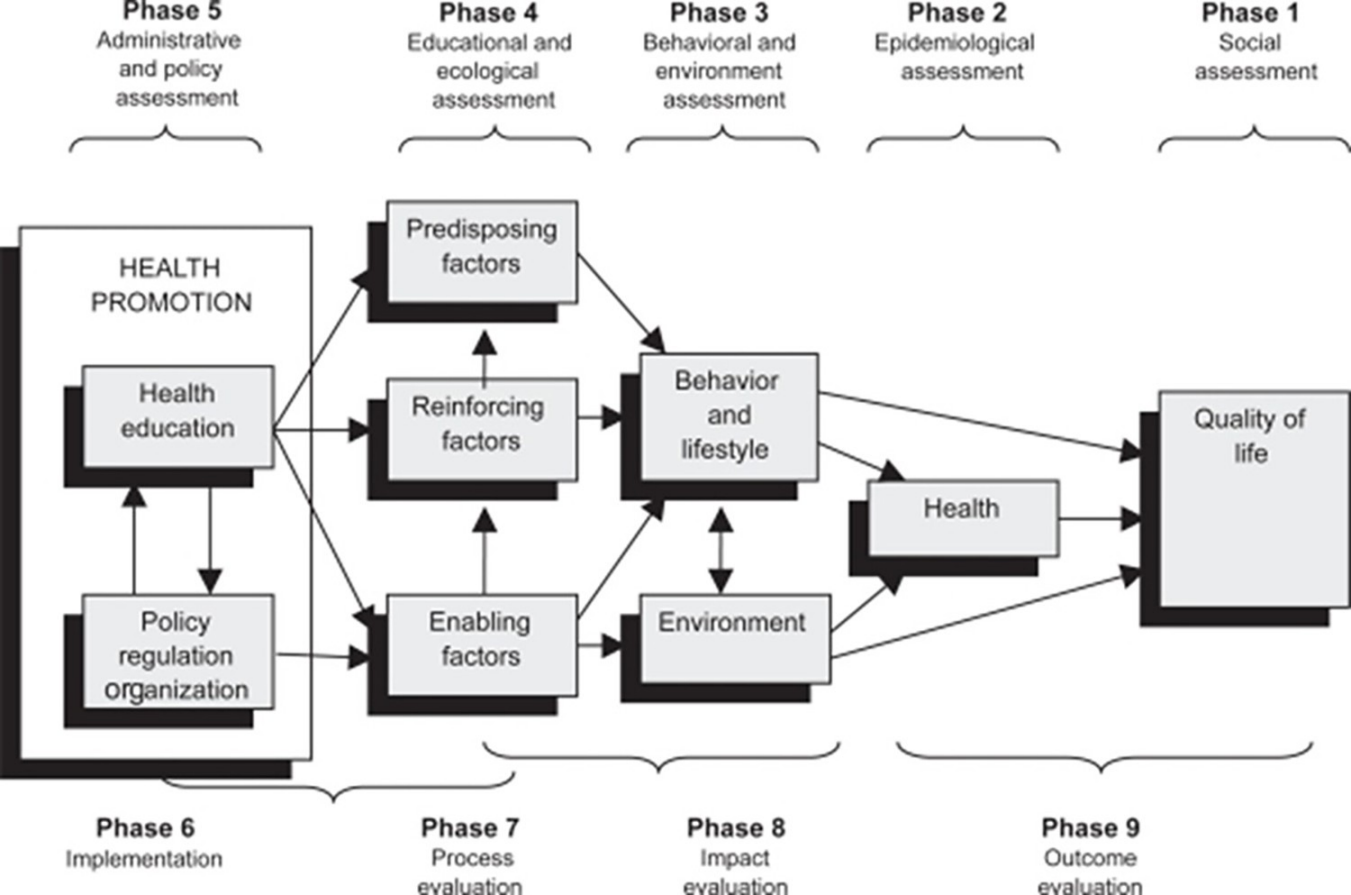
The PRECEDE-PROCEED model for health promotion, planning and evaluation [17].

Individualised semi-structured interviews were conducted to explore patients’ perceptions of PA, devised using the PRECEDE component of the PRECEDE-PROCEED model (Fig 2) [17]. Interviews were scheduled for a time and place convenient for participants and were conducted via telephone or face-to-face. Interviews were audio recorded using a Dictaphone (Sony-ICD-PX370, Sony Corporation, Japan) and transcribed verbatim for further data analysis.

#### 3.3.2. Phase 2

Phase 1 and 2 ran consecutively as findings from phase 1 informed the structure of phase 2. Phase two comprised of separate focus groups, the first included individuals with CF and their families, the second included CF MDT members. During phase 2 participants’ nominated family members were also invited to take part in the focus group. Focus group membership was determined by the participant and the term family was used to describe the individuals that participants deemed significant to invite and, in some cases, referred to friends as well as immediate relatives. Family members below the age of 14 were excluded as it was deemed that an appropriate level of maturity was required to comprehend and participate in the process [20]. The aim of the focus groups was to discuss the perceived barriers, facilitators and opportunities for PA participation and how this information could inform the development of a PA intervention. Individuals with CF attended separate focus groups in order to adhere to segregation procedures. Focus group membership was proposed to be between 3–8 individuals including a researcher, an individual with CF and their family. Where family members did not wish to participate a second semi-structured interview was conducted between the researcher and individual with CF, covering the same topics discussed during the focus groups. Members of the CF MDT participated in a separate focus group made up of MDT members and researchers, aiming for 3–8 members. All focus groups were audio recorded using a Dictaphone (Sony-ICD-PX370, Sony Corporation, Japan) and transcribed verbatim for further data analysis.

#### 3.2.3. Clinical measures

Medical notes were reviewed to obtained demographic data, anthropometric data, lung function and genotype for patients with CF. This data was used to describe the study sample in terms of demographics and disease severity.

### 3.3. Data analysis

Thematic analysis of transcripts was consistent with the six-stage process outlined by Braun & Clarke (2006) [21]. Transcripts were re-read to enable the researcher to become familiarised with the data and become immersed in the content. NVivo Pro 12 software package (QSR International Pty Ltd.,Doncaster, Victoria, Australia, 2019) was used to store and organise the transcripts. Data were coded in NVivo using first deductive then inductive analyses to capture latent meaning, adopting a constructivist approach to examine realities, meanings, and experiences of PA within a socio-economic framework. Initial themes were generated, reviewed and defined through discussion with a Health and Care Professions Council (HCPC) Registered Psychologist, with expertise using thematic analysis acting as a critical friend.

The constructed themes were clustered around the existing P-P model [17] and displayed using pen profiles centred around the key predisposing, enabling and reinforcing factors of the P-P model. Verbatim quotes are also used to illustrate findings where appropriate. Pen profiles provide a visual representation of data-sets via a diagram of key themes, a method previously used in similar research [22] and in line with the deductive framework provide by the P-P model [17]. Pen profiles were manually constructed from the transcribed data, with frequency count (square brackets) and verbatim quotes added to provide context. Trustworthiness and credibility were achieved through the following of robust guidelines and procedures for conducting thematic analysis. Consistent with the method and epistemological positioning this does not include traditional methods for assessing rigor such as member checking, inter-rater reliability or universal criteria but is achieved through appropriate transparency in reporting of epistemological stance, methodological approach, analytic procedures and presentation of themes and data [21,23].

## 4. Phase 1 results

### 4.1. Participant characteristics

Eleven participants (6 male) completed phase 1 of the study. Six were homozygous and four heterozygous for the Δ F508 mutation with one other mutation. Average age was 33.2 ± 7.1 years, Forced Expiratory Volume in one second (FEV_1_) was 2.06 ± 1.03 litres (54 ± 17%predicted) and Body Mass Index (BMI) was 22.5 ± 4.5.

### 4.2. Phase 1 –Individual interviews

Eleven semi-structured interviews were conducted. Interviews were audio recorded and transcribed verbatim. Interviews ranged from 25 minutes 45 seconds to 67 minutes 45 seconds in length, generating 8 hours 30 minutes of audio and 5,425 lines, 78,039 words of text. Average interview length, 42 minutes 22 seconds of audio, 493 lines, 7,094 words of text (Size 11 Calibri font with 1.15 line spacing).

Constructed themes are displayed using the principle predisposing, reinforcing, and enabling factors outlined in the PRECEDE component of the P-P model [17]. The analysis is represented using pen profiles.

#### 4.2.1. Predisposing factors

Predisposing factors include knowledge, attitudes, beliefs and perceived abilities. These factors may predispose a given health-related behaviour, in this case increase the likelihood of an individual engaging in PA [17].

**Fig 3:**
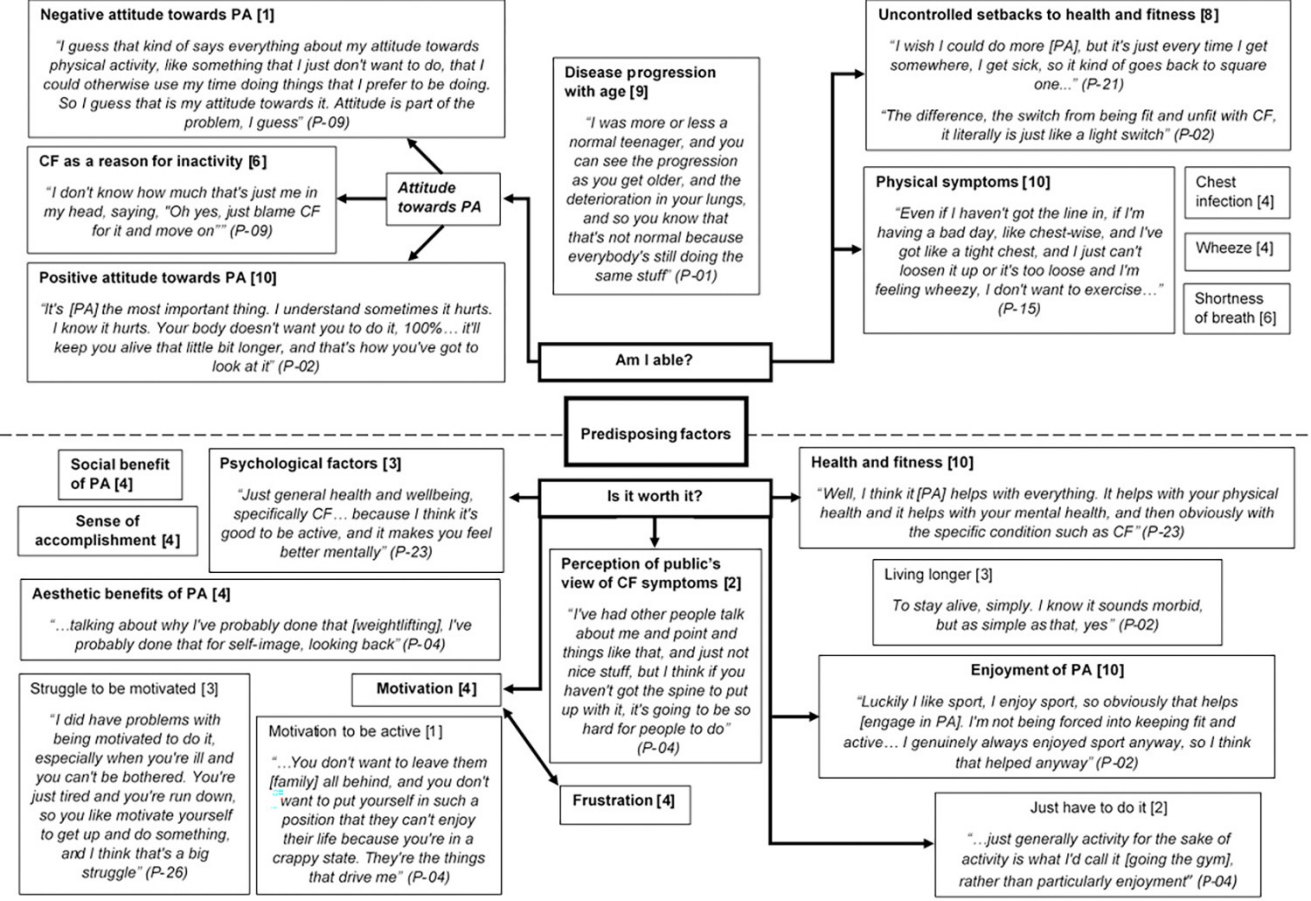
Pen profile displaying predisposing factors (n = 11) with illustrative quotes, associated participant number and frequency count provided in square brackets.

The principle predisposing factors discussed during the phase 1 interviews are organised into constructed themes and displayed as a pen profile (Fig 3). Themes are divided into two higher order themes *‘Is it worth it*?’ and *‘Am I able*?*’*, and further divided into five and four sub-groups respectively.

*4*.*2*.*1*.*1*. *Is it worth it*?. The ‘Is it worth it?’ higher order theme is related to the benefits and costs associated with PA, this includes attitudes, beliefs and enjoyment of PA.

**4.2.1.1.1. Enjoyment.** Enjoyment was reported as a key predisposing factor, with participants reporting that engaging in PA was easier as a result of enjoying activity. For example;

“*Luckily I like sport*, *I enjoy sport*, *so obviously that helps [to engage in PA]*. *I’m not being forced into keeping fit and active*… *I genuinely always enjoyed sport anyway*, *so I think that helped” (Partcipant-02*, *lines 94–97)*.

With participants expressing a preference for activities that they perceive to be more enjoyable;

“*I think everyone hates the gym*… *Just running on a treadmill*, *it’s just boring*. *Do something like fun like a class or something*, *that makes you forget you’re working out” (Partcipant-26*, *lines 280–282)*.

However, in some cases participants reported that regardless of whether or not they enjoyed activity it was necessary to engage in PA;

“*…just generally activity for the sake of activity is what I’d call it [using a gym] rather than particularly enjoyment” (Partcipant-09*, *lines 136–137)*.

**4.2.1.1.2. Health and Fitness.** Other factors reported related to the perceived improvements to health and fitness, frequently described as feeling better or living longer, with participants also aware of the negative consequences of inactivity for health, collectively these factors were coded as ‘health and fitness’ (Fig 3). For example;

“*Because you feel better [following exercise]… not just CF-wise*, *but in general” (Partcipant-30*, *line 91)*.

“*To stay alive*, *simply*. *I know it sounds morbid*, *but as simple as that” (Partcipant-02*, *line 241)*.

“*Yes*, *because I think my health could be a lot worse*, *really*, *if I don’t do all those things [PA]” (Partcipant-28*, *line 19)*.

**4.2.1.1.3. Psychological factors.** Participants also reported a number of psychological factors, with some describing a ‘mental aspect’ to engaging in PA or a perceived ‘mental benefit’ from engaging in PA.

“*When you’re not in a good place mentally*, *you can’t drive yourself to do things like go to the gym*, *because you’ve still got to drive yourself to just do things like take your meds*, *so it’s like one extra thing that you’ve got to do when you’re in a poor mental state” (Partcipant-04*, *lines 164–166)*.

“*Just general health and wellbeing*, *specifically CF*, *and just generally as well*, *because I think it’s good to be active*, *and it makes you feel better mentally” (Partcipant-23*, *lines 92–93)*.

Some factors relating to the participants’ beliefs about the benefits of PA were also considered to have an influence psychologically and formed a cluster (Fig 3). The factors forming sub-themes included a perceived ‘social’ benefit from engaging in PA, a desire to improve ‘aesthetically’, whether that be increased muscle mass and size or an improvement in shape and weight loss and finally engaging in PA to gain a ‘sense of accomplishment’ or achievement.

“*it’s [group based resistance training] a social thing as well for me*, *exercise*, *so I would have felt like I was missing out on that if I didn’t go [to a session]” (Partcipant-23*, *lines 64–66)*.

“*When you’re talking about why I’ve probably done that [weightlifting]*, *I’ve probably done that for self-image*, *looking back” (Partcipant-04*, *lines 227–228)*.

“*I love seeing an improvement in getting stronger as well*. *I like being able to look at the weights I couldn’t lift a month ago” (Partcipant-15*, *lines 163–164)*.

Participants in the current study also reported a lack of motivation as a barrier to PA:

“*I did have problems with being motivated to do it [PA]*, *especially when you’re ill and you can’t be bothered*. *You’re just tired and you’re run down*, *so you like motivate yourself to get up and do something*, *and I think that’s a big struggle*” *(Partcipant-26*, *lines 110–112)*.

One participant described how their motivation to lead a full and healthy life was also their motivation to engage in PA.

“*You don’t want to leave them all behind [family]*, *and you don’t want to put yourself in such a position that they can’t enjoy their life because you’re in a [poor] state*. *They’re the things that drive me” (Partcipant-04*, *lines 357–359)*.

A separate but related sub-theme was coded as ‘frustration’. Participants reported becoming demoralised when trying to achieve a desired level fitness, particularly following a period of exacerbation or admission. Other sources of frustration and disappointment included injuries, lack of exercise capacity or perceived poor performance. For example;

“*I know I’m not the worst [runner]*, *but also I get a bit frustrated that I should be able to get to the top of that hill*, *and I can’t get there*, *or I’m slowing down*, *so I have to come to a stop*, *as opposed to a slower pace” (Partcipant-03*, *lines 87–89)*.

This participant also expressed how they would like to know what their performance could be like if they were not limited by their CF. Highlighting an awareness that CF could limit PA but not to an extent that meant participating was not possible.

*I’d love to know how good a runner I could be if I didn’t have CF (Partcipant-03*, *line 146)*.

*4*.*2*.*1*.*2*. *Am I able*?. The ‘Am I able?’ higher order theme is concerned with perceived competence and self-efficacy for PA.

**4.2.1.2.1. Attitude towards physical activity.** PA was valued as important with participants demonstrating a positive attitude towards PA.

“*It’s the most important thing*. *I understand sometimes it hurts*. *I know it hurts*. *Your body doesn’t want you to do it*, *100%*… *it’ll keep you alive that little bit longer*, *and that’s how you’ve got to look at it” (Partcipant-02*, *lines 194–198)*.

It was also evident that a negative attitude towards PA could be detrimental to PA behaviour, even when individuals perceive PA to be important. For example;

“*I guess that kind of says everything about my attitude towards physical activity*, *like something that I just don’t want to do*, *that I could otherwise use my time doing things that I prefer to be doing*. *So I guess that is my attitude towards it*. *Attitude is part of the problem*, *I guess” (Partcipant-09*, *lines 110–113)*.

Participants suggested that CF provided an opportunity to use the condition as an excuse to not engage in PA. It was also evident that ambiguity exists between when CF presents real barriers to PA and when it serves as a convenient excuse. For example;

“*And I’ve seen it for myself*, *a lot of CF patients use it as an excuse or something*, *and I don’t like that*. *It’s not an excuse*, *it’s just a different way of living” (Partcipant-02*, *lines 180–181)*.

“*I don’t know how much that’s just me in my head*, *saying*, *"Oh yes*, *just blame CF for it and move on"” (Partcipant-09*, *lines 306–307)*.

**4.2.1.2.2. Progression with age.** The ability to engage in PA changes across the life course of an individual with CF, with participants describing how they have adjusted their activities and their expectations of what they could engage in as their disease has progressed.

“*I was more or less a normal teenager*, *and you can see the progression as you get older*, *and the deterioration in your lungs*, *and so you know that that’s not normal because everybody’s still doing the same stuff…” (Partcipant-01*, *lines 132–134)*.

**4.2.1.2.3. Physical symptoms.** Many of the barriers to PA described were real physical symptoms rather than perceived factors. Despite a willingness to engage in PA, the physical symptoms described could make this challenging. For example;

“*Even if I haven’t got the line in*, *if I’m having a bad day*, *like chest-wise*, *and I’ve got like a tight chest*, *and I just can’t loosen it up or it’s too loose and I’m feeling wheezy*, *I don’t want to exercise*, *so I will stay in bed*.*” (Partcipant-15*, *lines 110–112)*.

“*It’s a struggle sometimes when you’re walking round*. *So you get out of breath quite easily*, *and you’ll get tight-chested quite often*. *It’s a bit horrid*, *yes” (Partcipant-28*, *lines 7–8)*.

**4.2.1.2.4. Uncontrolled set-backs.** Participants accepted that managing the symptoms of CF was inherent to the condition, though the difficultly of managing the unpredictable and uncontrollable nature of the set-backs was a source of frustration. Set-backs were often described as part of a cycle of improvements in health and fitness followed by a set-back undoing those improvements.

“*I wish I could do more*, *but it’s just every time I get somewhere*, *I get sick*, *so it kind of goes back to square one…” (Partcipant-21*, *lines 114–115)*.

“*The difference*, *the switch from being fit and unfit with CF*, *it literally is just like a light switch” (Partcipant-02*, *lines 248–24*

#### 4.2.2. Enabling factors

**Fig 4:**
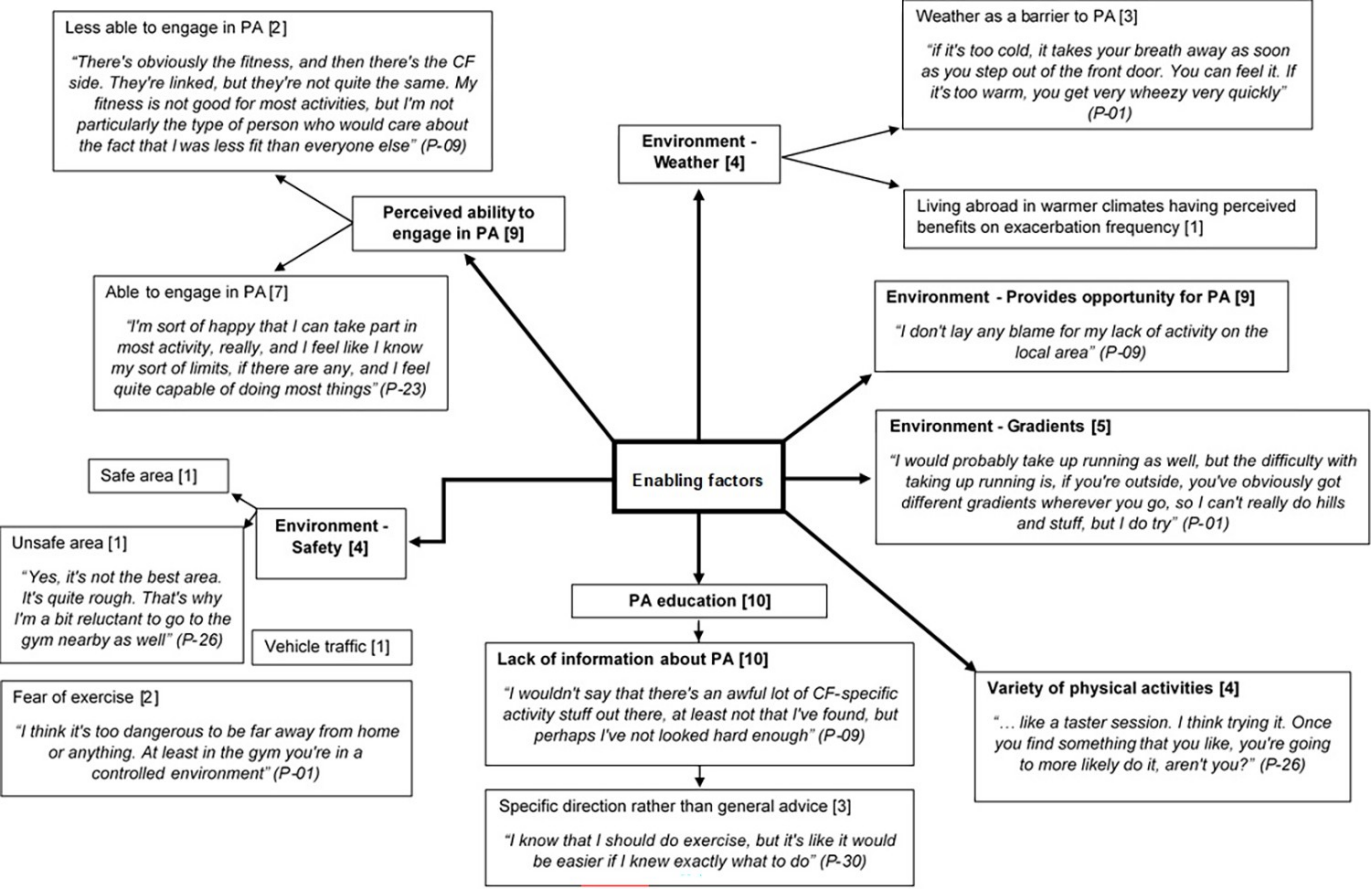
Pen profile displaying enabling factors (n = 11) with illustrative quotes, associated participant number and frequency count provided in square brackets.

Enabling factors represent the immediate targets for interventions as they represent the new skills and/or organisational changes required to allow engagement in PA [17]. Enabling factors can include environmental conditions such as the availability and accessibility of resources, conditions of living such as childcare arrangements or transport, weather and safety [17]. Enabling factors also relate to the necessary skills required to complete a behaviour. Enabling factors are presented in Fig 4.

*4*.*2*.*2*.*1*. *Environmental opportunity for physical activity*. The participants in the current study were recruited from a CF centre providing care for a large geographical area, with some patients living ~150 miles from clinic. Despite this, participants across different geographical areas reported having access to resources and facilities to allow them to engage in PA.

“*I don’t lay any blame for my lack of activity on the local area” (Partcipant-09*, *line 297–298)*.

The physical environment and terrain were reported as barriers to PA. Although this is not a factor that could be modified as part of an intervention, it may require consideration.

“*…I would probably take up running as well*, *but the difficulty with taking up running is*, *if you’re outside*, *you’ve obviously got different gradients wherever you go*, *so I can’t really do hills and stuff*, *but I do try…” (Partcipant-01*, *lines 198–200)*.

*4*.*2*.*2*.*2*. *Safety*. Factors relating to safety were also constructed as a theme. Whilst one participant felt that the local area was a safe place to engage in PA they also expressed concerns about exercising whilst alone and potentially in isolation in the event of exercise induced symptoms which led them to only engage in PA in controlled environments such as a gym. This concern was echoed by participants describing a level of fear of exercise. For example;

“*You don’t feel at risk at all here” (Partcipant-01*, *line 411)*.

“*…I think it’s too dangerous to be far away from home or anything*. *At least in the gym you’re in a controlled environment” (Partcipant-01*, *lines 214–215)*.

“*…I was a bit worried at first*, *and I was getting really out of breath*, *and I didn’t know if my lungs could handle…I didn’t know how much they could handle…” (Partcipant-21*, *lines 183–185)*.

Not all participants deemed their local environment as safe and stated this as a barrier to their activity, although it was a barrier that they could negotiate by finding alternative activities.

“*Yes*, *it’s not the best area*. *It’s quite rough*. *That’s why I’m a bit reluctant to go to the gym nearby as well…” (Partcipant-26*, *lines 228–229)*.

“*… I don’t like to actually see people running alongside traffic*, *and there’s a motorway by me as well*, *so there can be an awful lot of pollution and stuff*, *and I know we can’t see it*, *but it’s always in the back of my mind as well” (Partcipant-04*, *lines 537–540)*.

*4*.*2*.*2*.*3*. *Weather*. Weather conditions were also reported as factor affecting PA and general well-being of participants and were coded as a sub-theme containing both positive and detrimental aspects.

“*…because I was abroad*, *you know*, *the temperature and the weather was different*, *and I barely ever got sick*, *and I was only admitted like once every couple of years” (Partcipant-26*, *lines 218–220)*.

“*…if it’s too cold*, *it takes your breath away as soon as you step out of the front door*. *You can feel it*. *If it’s too warm*, *you get very wheezy very quickly…” (Partcipant-01*, *lines 217–219)*.

*4*.*2*.*2*.*4*. *Perceived ability*. Having the skills required to participate in PA is considered a necessary determinant of PA. Participants acknowledge that their ability to take part in certain activities may be limited, however there was an awareness of such limitations, with participants perceiving themselves as able to engage in PA. For example;

“*I’m sort of happy that I can take part in most activity*, *really*, *and I feel like I know my sort of limits*, *if there are any*, *and I feel quite capable of doing most things” (Partcipant-23*, *lines 223–224)*.

For some these limitations were perceived to negatively affect their ability to engage in PA.

“*There’s obviously the fitness*, *and then there’s the CF side*. *They’re linked*, *but they’re not quite the same*. *My fitness is not good for most activities*, *but I’m not particularly the type of person who would care about the fact that I was less fit than everyone else*.*” (Partcipant-9*, *lines 300–302)*.

Having the skills necessary to engage in PA was not always related to physical ability but rather to having a knowledge of PA. Participants reported a lack of information about PA for individuals with CF, stating that specific guidance would help to facilitate their PA. For example;

“*…I wouldn’t say that there’s an awful lot of CF-specific activity stuff out there*, *at least not that I’ve found*, *but perhaps I’ve not looked hard enough” (Partcipant-09*, *lines 326–328)*.

“*I know that I should do exercise*, *but it’s like it would be easier if I knew exactly what to do…” (Partcipant-30*, *lines 239–240)*.

#### 4.2.3. Reinforcing factors

Reinforcing factors relate to the consequences of a behaviour and whether individuals receive positive or negative feedback or social support for the behaviour [17]. In the current study these are factors that reinforce PA behaviour and could include the influence of peers and family either directly or indirectly.

**Fig 5:**
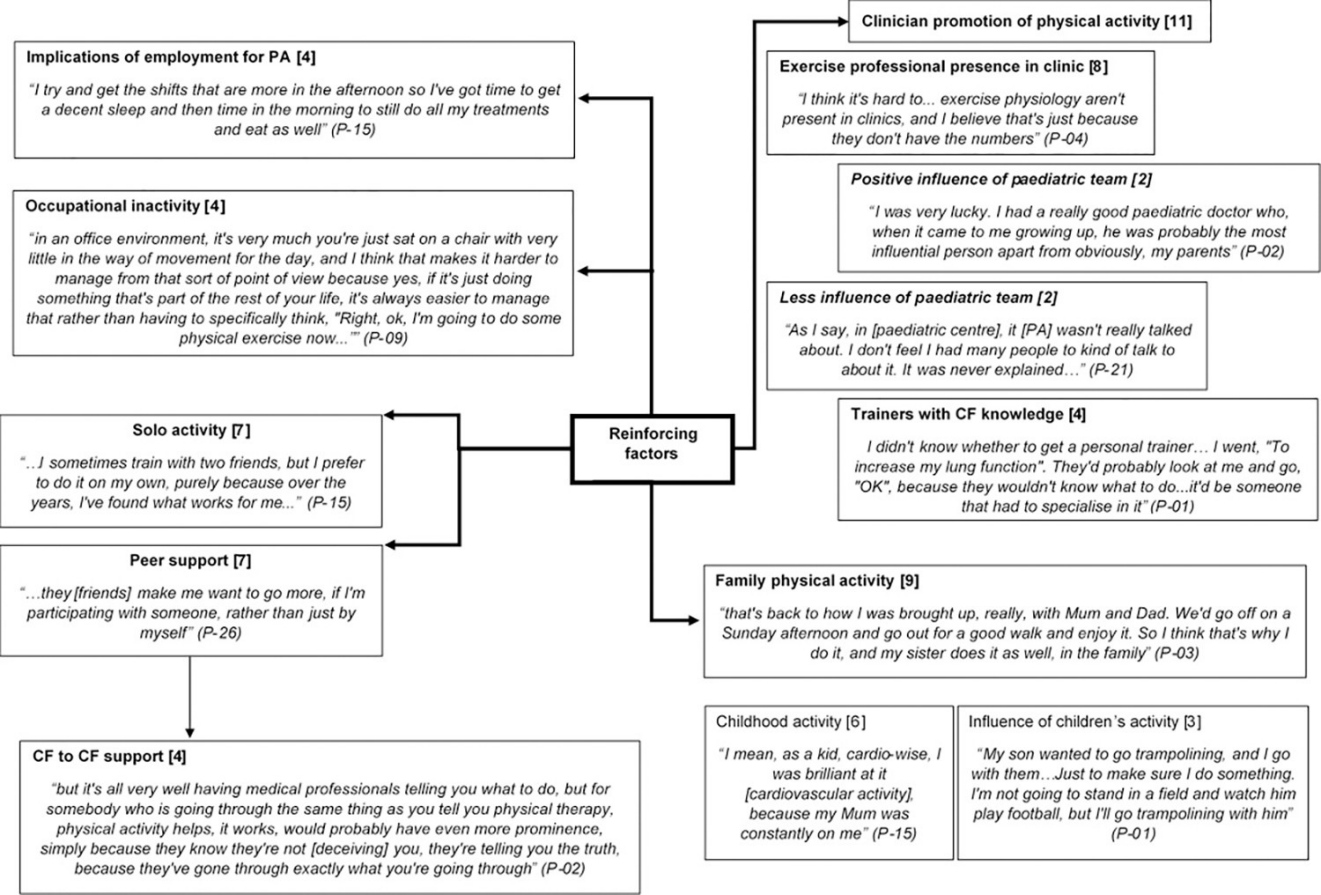
Pen profile displaying reinforcing factors (n = 11) with illustrative quotes, associated participant number and frequency count provided in square brackets.

*4*.*2*.*3*.*1*. *Clinician promotion of physical activity*. Within reinforcing factors, the influence of clinical teams was constructed as a higher order theme with a number of sub-themes (Fig 5). The first of which was the presence of an exercise professional at routine CF clinics as part of the CF multi-disciplinary team. The benefit of having access to an exercise team was recognised, although participants were also aware that the service could be limited.

“*I do think the exercise team or someone with an exercise background should be part of the team on every visit” (Partcipant-15*, *lines 461–462)*.

“*I think it’s hard to*…*exercise physiology aren’t present in clinics*, *and I believe that’s just because they don’t have the numbers” (Partcipant-04*, *lines 468–469)*.

Some participants also reflected on their experience within paediatric centres and the influence this had on their subsequent PA behaviour throughout their life. These experiences were mixed, with some participants grateful for receiving *‘good’* paediatric care and being encouraged to be physically active, whilst others felt that there was a lack of promotion of PA during their paediatric care.

“*I was very lucky*. *I had a really good paediatric doctor who*, *when it came to me growing up*, *he was probably the most influential person apart from obviously*, *my parents…” (Partcipant-02*, *lines 198–200)*.

“*When I was in [paediatric centre] it [PA] was never a big thing back then*, *though*. *You know*, *exercise*, *and it was there*, *but it was never promoted*, *do you know what I mean*? *and encouraged” (Partcipant-21*, *lines 222–223)*.

Participants reported seeking out support in their local community from gyms or leisure centres, however participants felt that exercise professionals outside of CF care such as personal trainers would need an understanding of CF to be able to provide effective exercise programmes and work with individuals with CF.

“*I didn’t know whether to get a personal trainer…I went*, *"To increase my lung function"*. *They’d probably look at me and go*, *"OK"*, *because they wouldn’t know what to do*…*it’d be someone that had to specialise in it” (Partcipant-01*, *lines 549–554)*.

*4*.*2*.*3*.*2*. *Family physical activity*. Through the course of the research the participants self-defined the term family. Participants varied in age (25–42 years), marital status (n = 6 single/never married) and living arrangements (parental home, rented accommodation, home owner), with some participants having children of their own (n = 3). Therefore, the term family relates to the individuals that participants perceived to be significant within their lives, either at present or during their childhood, this may include a spouse/partner, child, sibling and/or parent/carer. Separate themes were constructed for activity as a child, and the influence of children on activity. The family were perceived to have significant influence on PA behaviour, with participants describing positive experiences engaging in PA as a child. Having their own children was also described as a positive influence on participants’ own PA.

“*…that’s back to how I was brought up*, *really*, *with Mum and Dad*. *We’d go off on a Sunday afternoon and go out for a good walk and enjoy it*. *So I think that’s why I do it*, *and my sister does it as well*, *in the family” (Partcipant-03*, *lines 131–133)*.

“*My son wanted to go trampolining*, *and I go with them…Just to make sure I do something*. *I’m not going to stand in a field and watch him play football*, *but I’ll go trampolining with him” (Partcipant-01*, *lines 178–190)*.

*4*.*2*.*3*.*3*. *Peer support*. Peer support and solo PA were constructed as separate themes and given equal attention. Whilst participants described engaging in PA with others at various stages in their lives and perceiving motivational benefits of this, they also described engaging in a large proportion of their PA alone and preferring this.

“*…they [friends] make me want to go [to the gym] more*, *if I’m participating with someone*, *rather than just by myself” (Partcipant-26*, *lines 162–163)*.

“*I sometimes train with two friends*, *but I prefer to do it on my own*, *purely because over the years*, *I’ve found what works for me…” (Partcipant-15*, *lines 181–182)*.

Aside from actually engaging in PA with each other participants perceived that being able to communicate with individuals deemed *‘like them’* would serve as support and reinforce positive PA behaviours. Participants also stated that the support would be best coming from someone in a similar position to them in terms of fitness, disease severity and lung function.

“*…but it’s all very well having medical professionals telling you what to do*, *but for somebody who is going through the same thing as you tell you physical therapy*, *physical activity helps*, *it works*, *would probably have even more prominence*, *simply because they know they’re not [deceiving] you*, *they’re telling you the truth*, *because they’ve gone through exactly what you’re going through” (Partcipant-02 lines*, *513–518)*.

*4*.*2*.*3*.*4*. *Employment*. Of the participants in the current study 8 (73%) were employed in full- or part-time work. Work commitments presented challenges for managing CF, with participants reporting a requirement to have flexible arrangements in order to manage their CF, in some cases this included reducing hours from full- to part-time or leaving work all together.

“*…I try and get the shifts that are more in the afternoon so I’ve got time to get a decent sleep and then time in the morning to still do all my treatments and eat as well” (Partcipant-15*, *lines 49–51)*.

Whilst for some employment offered the opportunity to be physically active throughout working hours for others employment facilitated large periods of inactivity. For example;

“*Well*, *I was on my feet all day at work*…*we’re on our feet quite a lot*. *I don’t sit down at a computer*, *I don’t sit down at a desk*. *I’m standing and walking up and down…all day*, *lifting*, *carrying heavy boxes*, *putting away stock” (Partcipant-03*, *lines 51–54)*.

“*…in an office environment*, *it’s very much you’re just sat on a chair with very little in the way of movement for the day*, *and I think that makes it harder to manage from that sort of point of view because yes*, *if it’s just doing something that’s part of the rest of your life*, *it’s always easier to manage that rather than having to specifically think*, *"Right*, *ok*, *I’m going to do some physical exercise now because I have to"…” (Partcipant-09*, *lines 49–54)*.

#### 4.2.4. Nuanced

**Fig 6:**
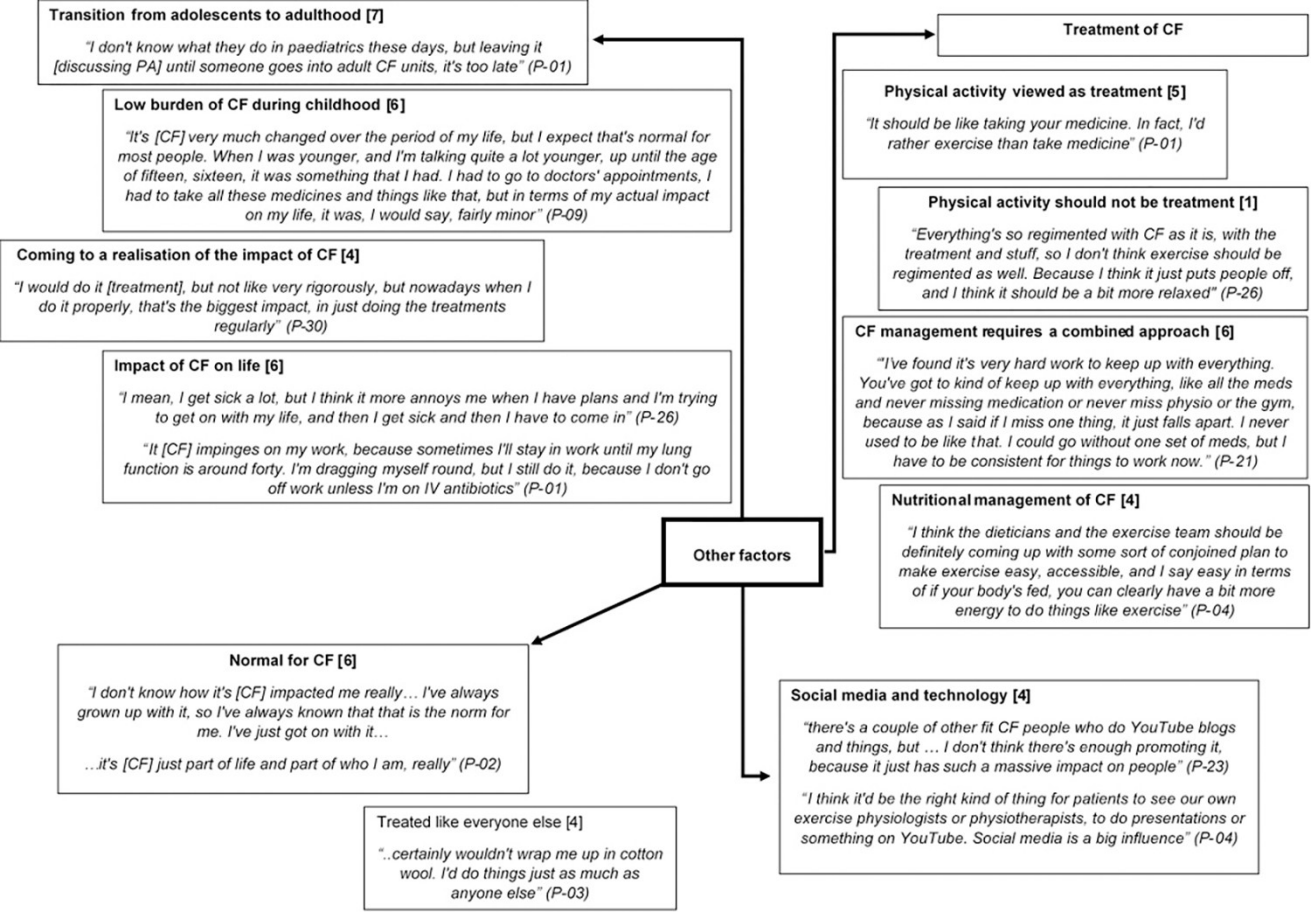
Pen profile displaying ‘other’ factors (n = 11) with illustrative quotes, associated participant number and frequency count provided in square brackets.

The P-P model provides a framework to explore health behaviours and whilst many of the themes discussed fit within this framework not all of the constructed themes did and are denoted as ‘other factors’. This inductive content analysis approach to thematic analysis is also consistent with theoretically-flexible reflexive thematic analysis [18]. These factors relate to transitioning from paediatric to adult care (transition), living with CF (normal for CF), the management of CF (treatment) and the role of social media and technology in the management of CF (Fig 6).

*4*.*2*.*4*.*1*. *Transition*. The majority of CF centres in the UK typically care for either adult or paediatric patients with a small number of combined centres. The transition process differs between centres but typically involves patients moving from receiving care within a paediatric centre to an adult centre and occurs around the age of 16–18 years of age [24]. A number of participants recognised this as an important period in their lives, during which they felt they would have benefitted from support around their PA. For example;

“*…I don’t know what they do in paediatrics these days*, *but leaving it [discussing PA] until someone goes into adult CF units*, *it’s too late” (Partcipant-01*, *lines 304–306)*.

The burden of CF during childhood was reported to be much lower, with participants describing that they were relatively well as a child but at a certain period (often as a teenager into early adulthood) CF began to have a greater impact on their life. Some also described that they came to a stark realisation that CF was serious and that it was going to have a significant impact on their life and require significant effort to manage. For example;

“*It’s [CF] very much changed over the period of my life*, *but I expect that’s normal for most people*. *When I was younger*, *and I’m talking quite a lot younger*, *up until the age of fifteen*, *sixteen*, *it was something that I had*. *I had to go to doctors’ appointments*, *I had to take all these medicines and things like that*, *but in terms of my actual impact on my life*, *it was*, *I would say*, *fairly minor” (Partcipant-9*, *lines 10–14)*.

“*…I would do it [treatment]*, *but not like very rigorously*, *but nowadays when I do it properly*, *that’s the biggest impact*, *in just doing the treatments regularly…” (Partcipant-30*, *lines 35–37)*.

Participants also described the impact CF can have on their lives as an adult.

“*I mean*, *I get sick a lot*, *but I think it more annoys me when I have plans and I’m trying to get on with my life*, *and then I get sick and then I have to come in…” (Partcipant-26*, *lines 18–19)*.

“*It [CF] impinges on my work*, *because sometimes I’ll stay in work until my lung function’s around forty [%predicted]*. *I’m dragging myself round*, *but I still do it*, *because I don’t go off work unless I’m on IV antibiotics” (Partcipant-01*, *lines 195–197)*.

*4*.*2*.*4*.*2*. *Treatment*. Previous research demonstrates that the management of CF requires a complex and burdensome routine [1] and PA is often recommended in routine clinical care as part of the management of the condition. For study participants, PA was therefore perceived as a ‘treatment’ and likened to taking medication, with some describing how they ‘have to do it *[PA]*’.

“*It should be like taking your medicine*. *In fact*, *I’d rather exercise than take medicine” (Partcipant-01*, *lines 499–500)*.

Others felt that PA should not be perceived this way, instead it should be less structured and done for the purposes of fun and enjoyment rather than as treatment.

“*Everything’s so regimented with CF as it is*, *with the treatment and stuff*, *so I don’t think exercise should be regimented as well*. *Because I think it just puts people off*, *and I think it should be a bit more relaxed…” (Partcipant-26*, *lines 314–316)*.

Ultimately though the management of CF includes many factors, with participants describing how each aspect of their treatment required their efforts and that shortcomings in any one aspect could have consequences for the other aspects of their management and their health overall. For example;

“*I’ve found it’s very hard work to keep up with everything*. *You’ve got to kind of keep up with everything*, *like all the meds and never missing medication or never miss physio or the gym*, *because I said if I miss one thing*, *it just falls apart*. *I never used to be like that*. *I could go without one set of meds*, *but I have to be consistent for things to work now” (Partcipant-21*, *lines 37–40)*.

An example of the multi-faceted treatment of CF is that of nutritional management. Participants reported symptoms relating to digestion such as bloating and frequently needing to use the toilet as barriers to PA as well as stating that PA could improve some of these symptoms. It’s not surprising then that participants perceived PA and nutrition to be complementary to each other. For example;

“*I think the dieticians and the exercise team should be definitely coming up with some sort of conjoined plan to make exercise easy*, *accessible*, *and I say easy in terms of if your body’s fed*, *you can clearly have a bit more energy to do things like exercise” (Partcipant-04*, *lines 680–683)*.

*4.2.4.3. Social media and technology*. There is an increasing interest in the use of technology to support PA in health care [25] not only reported in the academic literature but also amongst participants. For example;

“*…there’s a couple of other fit CF people who do YouTube blogs and things*, *but…I don’t think there’s enough promoting it*, *because it just has such a massive impact on people” (Partcipant-23*, *line 258–260)*.

“*I think it’d be the right kind of thing for patients to see our own exercise physiologists or physiotherapists*, *to do presentations or something on YouTube*. *Social media is a big influence…” (Partcipant-04*, *lines 619–621)*.

*4*.*2*.*4*.*4*. *Normal for CF*. Finally, when discussing CF, participants stated that they didn’t know any different often using the phrase ‘‘it’s just normal for CF”. Participants accepted that their lives may be impacted by CF but that’s what is normal for them so they ‘just get on with it’ with an expectation to be treated like everyone else. For example;

“*…I don’t know how it’s impacted me really…I’ve always grown up with it*, *so I’ve always known that that is the norm for me*. *I’ve just got on with it…” (Partcipant-02*, *lines 64–67)*.

“*…it’s just part of life and part of who I am*, *really” (Partcipant-02*, *lines 222–223)*.

“*…certainly wouldn’t wrap me up in cotton wool*. *I’d do things just as much as anyone else…” (Partcipant-03*, *lines 23–24)*.

## 5. Phase 1 discussion

The aim of phase 1 was explore patients’ perceptions of PA and understand the ecological correlates of PA in adults with Cystic Fibrosis. The data obtained during this phase contributes to the social and epidemiological diagnosis as outlined in phase 1 and 2 of the P-P model [17].

The principle predisposing barriers related to participants physical and mental wellbeing, which manifested as both a barrier and a facilitator of PA behaviour. CF is characterised by a progressive decline in physical function, which for the participants presented as a number of challenging symptoms and set-backs. The findings of the current study are consistent with existing literature in that the range of symptoms reported includes numerous physical and psychosocial symptoms, reported with varied frequency and severity [1]. Participants perceived that PA had the potential to slow the rate of this decline and manage the symptoms associated with the condition. There is limited data available to assess the association between PA and symptom burden or HRQoL [26], although there is evidence to support an association between higher level of PA and reduced rate of decline in lung function [26,27]. Despite recognition of the potential benefits of PA, it appears that enjoyment is an important correlate of PA. This finding is consistent with findings from similar qualitative research exploring the perceptions of PA among children with CF, which also reported enjoyment as a facilitating factor for PA [28]. Lewis *et al*. suggests that interventions to promote PA in low-active adults should target increasing enjoyment first, which may in turn improve self-efficacy and motivation for PA [29]. Based on these findings it was recommended that practitioners should encourage individuals to engage in a variety of activities and promote enjoyment [29]. Motivation for PA was also reported as barrier to PA in the current study. It is well reported in the literature that motivation is an important determinant of PA behaviour [30], with a recent review of PA literature finding motivation, self-efficacy and self-regulation were consistently reported as correlates of PA [31]. Understanding of motivation is therefore important for informing the development of interventions to promote PA. The phased approach of the current research allowed for factors to be explored further in phase 2. Since disease progression and physical symptoms were not modifiable behaviours the focus of phase two was to understand the impact of these factors and to further explore the enjoyment of and motivation for PA.

The principle enabling factors related to participants having the skills necessary to engage in PA. Higher self-efficacy for PA and the perceived ease of activity has previously been shown to be associated with increased levels of PA [32]. Participants reported having the skills necessary to engage in PA but felt that additional support and further direction would be beneficial to enhance PA participation. Whilst the environment offered opportunity to engage in PA, safety (of the environment) and fear of limitations to exercise were reported as factors that may require consideration. Whilst the fear of exercise induced complications was not universal in participants in the current study it is well recognised in other populations such as patients attending cardiac rehabilitation and individuals with Type I diabetes [33,34]. Following a programme of exercise-based cardiac rehabilitation the fear of exercise reduced in participants, likewise a programme of support and education is recommended to reduce the fear of hypoglycaemic events during exercise in individuals with Type I diabetes [33,34]. The safety concerns in the current study also related to environmental factors such as traffic and pollution.

The presence of health care professionals with a special interest in PA and exercise within CF MDTs and clinics was reported as a key reinforcing factor for PA behaviour. The family also play a role in reinforcing PA behaviour in both childhood and adulthood. Similar research with families of children with and without CF also recommend involving the family in PA promotion, providing education for parents and incorporating PA into familial daily lifestyle [35,36]. Understanding the roles of the clinical team and families in delivering a PA intervention was therefore integrated into the phase 2 focus groups schedules.

The transition process, during adolescence and early adulthood was reported as an important period in the life of an individual with CF. This period is also associated with a reduction in PA in the general population [37], highlighting the need for additional support during this period, particularly for individuals with CF for whom the transition period can present a number of additional challenges [38]. This period was therefore explored further during the phase 2 focus groups, with the aim of determining when the most appropriate time to implement a PA intervention would be. Additionally, social media and technology were perceived to be influential for PA and were also explore further during phase 2.

The focus groups were designed to identify the modifiable behavioural characteristics associated with each of the factors identified in phase 1 in order to set achievable objectives for an intervention to promote PA in adults with CF. The constructed themes were interpreted based on; 1) Who an intervention should target 2) What action or change is required 3) To what extent an improvement in health outcomes can be expected 4) When the behaviour should be targeted. In keeping with an action research design, factors that could be modified were prioritised over factors in which modifiable action could not be taken.

## 6. Phase 2 results

Nine semi-structured focus groups were conducted with an individual with CF who also participated in phase 1, nominated members of their family and a researcher. Focus groups were audio recorded and transcribed verbatim. Focus groups ranged from 44 minutes 45 seconds to 64 minutes 15 seconds in length, generating 8 hours 30 minutes of audio and 6,221 lines, 87,558 words of text. Average interview length, 56 minutes 38 seconds of audio, 691 lines, 9,729 words of text (Size 11 Calibri font with 1.15 line spacing).

An additional semi-structured focus group was conducted with three members of the CF care team (2 consultants and 1 exercise physiologist) and a researcher. Generating 48 minutes of audio and 652 lines, 9372 words of text (Size 11 Calibri font with 1.15 line spacing).

During each focus group the principle predisposing, reinforcing, enabling and nuanced themes identified during phase 1 were discussed to inform the development of the objectives of an intervention to promote PA in adults with CF. The collective results from both the clinician and patient focus groups are discussed below, with areas of agreement/disagreement highlighted.

### 6.1. Participant characteristics

Nine participants with CF (5 male) completed phase two of the study. Average age was 32.8 ± 7.0 years, FEV_1_ was 2.07 ± 1.08 litres (52 ± 18%predicted) and BMI was 22.9. ± 5.0.

### 6.2. Overall aim of an intervention to increase PA in individuals with CF

The primary aim of an intervention was reported as improving the overall ‘wellbeing’ of individuals with CF. For some participants this meant improvements in key outcome measures (Lung function, exacerbation frequency, fitness or expectoration), for others this meant an ability to perform activities of daily living without limitation. A second aim was simply to increase PA, likely in view of this being associated with positive health outcomes. A final aim was to be able to engage individuals with CF a programme and to keep them motivated to engage in PA.

“*Well*, *obviously*, *increased physical activity*, *but yes…It has to be working with the patient to find something that they’re going to do*, *basically*, *and that sounds really stupid and obvious*, *but*…*You’ve got to ease yourself into it…” (Partcipant-09*, *lines 465–471)*.

### 6.3. Outcomes

**Fig 7:**
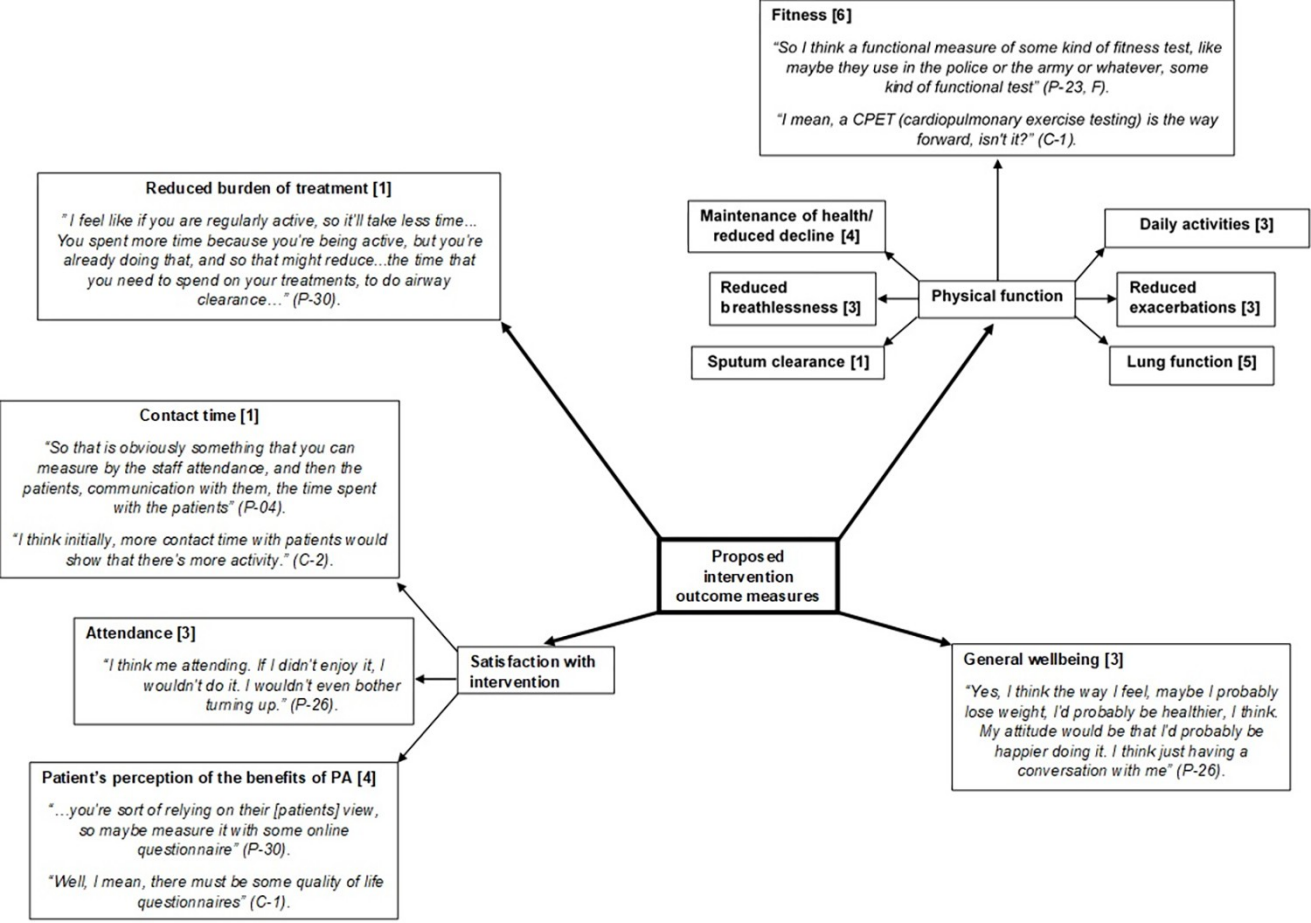
Pen profile displaying participant (patients (n = 9) & clinicians (n = 3) selected intervention outcome measures with illustrative quotes, associated participant number and frequency count provided in square brackets.

Consistent with phases 3 and 4 of the P-P model [17] the focus group data was used to help create themes to create measurable behavioural and environmental objectives which could ultimately be used to determine the success of an intervention (Fig 7).

Participants reported that an improvement in how they felt would be an important outcome to determine the success of an intervention. For example:

“*Yes*, *I think the way I feel*, *maybe I probably lose weight*, *I’d probably be healthier*, *I think*. *My attitude would be that I’d probably be happier doing it*. *I think just having a conversation with me” (Partcipant-26*, *lines 397–399)*.

Whilst participants perceived that the way they felt and general wellbeing were important they also acknowledged that this may not be a measurable outcome:

“*…I know myself [how I feel]*, *but I don’t know how you would capture it” (Partcipant-23*, *lines 424–425)*.

A proposed measure to reflect general wellbeing was the use of a questionnaire to assess self-reported quality of life:

“*…you’re sort of relying on their [patients] view*, *so maybe measure it with some online questionnaire” (Partcipant-30*, *lines 332–333)*.

“*Well*, *I mean*, *there must be some quality of life questionnaires” (Clinician-01*, *line 382)*.

There were a number of outcome measures reported as themes which related to improved physical function such as lung function, reduced exacerbations, reduced breathlessness and improved sputum clearance that corresponded to the predisposing factors identified during phase 1. This physical function can be reflected in a measure of ‘functional capacity’ or fitness. Participants recognised that a functional measure could reflect their ability to complete activities of daily living and meet the demands of their environment. Clinicians also recognised that a measure of functional capacity could reflect ‘overall robustness’ of patients. For example:

“*So I think a functional measure of some kind of fitness test*, *like maybe they use in the police or the army or whatever*, *some kind of functional test” (Partcipant-23*, *F*, *lines 363–364)*.

“*it’s just being able to do everything that you want to in life without feeling ill and tired” (Partcipant-23*, *lines 549–550)*.

“*I mean*, *a CPET (cardiopulmonary exercise test) is the way forward*, *isn’t it*?*” (Clinician-01*, *line 374)*.

The final outcomes reported related to the overall satisfaction with an intervention and willingness to engage with PA. Attendance was suggested as a measure of engagement;

“*I think me attending*. *If I didn’t enjoy it*, *I wouldn’t do it*. *I wouldn’t even bother turning up” (Partcipant-26*, *line 393)*.

As was contact time between individuals with CF and a member of the clinical team responsible for the promotion of PA;

“*So that is obviously something that you can measure by the staff attendance*, *and then the patients*, *communication with them*, *the time spent with the patients” (Partcipant-04*, *line 551–512)*.

“*I think initially*, *more contact time with patients would show that there’s more activity” (Clinican-02*, *line 633)*.

### 6.4. Intervention design

In order to further explore how these objectives could be achieved the design of the intervention was discussed, with focus on who would be responsible for managing the intervention, where the intervention could take place and what the intervention would look like.

#### 6.4.1. Intervention design–Who is responsible for managing a PA intervention?

Individuals reported as reinforcing PA during phase 1 were also reported as individuals important to the development of an intervention. This included the presence of an exercise professional within the CF MDT, engaging the family and individuals with CF themselves. Individuals with CF and their families perceived that the success of an intervention would require the whole CF care team to view PA with the same importance as other treatments, going on to state that this would have to start with the consultant. For example:

“*This is lots of little nudges to change the course of their behaviour*, *and that’s just every clinician*, *nurse*, *physio*, *one of us when we [clinical team] see them [patients]” (Clinician-02*, *lines 549–550)*.

“*So I think it [PA promotion] has to stem from the consultant or doctor*, *that firstly they see physiotherapy and physical activity as high up as medicine” (Partcipant-01*, *lines 566–567)*.

Within the wider team an exercise physiologist was identified as the key individual who would be responsible for the day-to-day patient contact and reinforcing the promotion of PA. As demonstrated below:

“*Yes*, *from day one…you’ve got to have a team of physiotherapists*, *physiologists that fully understand” (Partcipant-01*, *lines 571–572)*.

This quote also highlights the requirement for the intervention to be delivered by individuals with a knowledge and understanding of CF, an opinion shared by both individuals with CF and clinicians. Despite this there is not a defined qualification, training pathway or accreditation for exercise professionals to specialise in CF.

“*…they know CF and they’re sympathetic to your needs and the demands of the condition*. *That’s what I feel*. *It needs to come from a CF-based background” (Partcipant-03*, *lines 584–585)*.

“*somebody with some experience and a real interest in it [CF]” (Clinician-02*, *line 595)*.

Involvement of a psychologist as part of an intervention was also perceived as important, owing to the psychological barriers to PA discussed during phase 1;

“*Because it is a huge amount of that [barriers to PA]*, *it is all in your head*, *so perhaps someone who deals in your head will help” (Partcipant-09*, *lines 400–401)*.

There was a belief that the care team could, in effect, become ‘family by proxy’ in the absence of family support but individuals with family support felt that they should be involved in any interventions to promote PA and in their CF care more broadly. For example:

“*What we should be doing is having the parents gradually support you*, *and if you haven’t got parents*, *you need peers around you*. *Maybe it’s guardians*, *maybe it’s friends*, *or the CF team” (Partcipant-04*, *lines 154–156)*.

#### 6.4.2. Intervention design–Where should an intervention take place?

Findings from phase 1 indicated that participants did not perceive environmental factors to be a barrier to PA *per se*. The hospital/clinic, home, wider community and internet were identified as potential locations for a PA based intervention to take place. The hospital represented a familiar and convenient location, as well as providing access to health care professionals.

“*I think practically*, *it would probably be in clinic” (Partcipant-30*, *line 237)*.

The home was also perceived as suitable location for a PA intervention, particularly for individuals who may have limited functional capacity and/or feel less able to participate in PA. Although PA would be carried out away from the hospital, a level of support in the form of communication or an occasional home visit was deemed as an important component of the intervention. For example:

“*They could include just household-type stuff*, *like perhaps ten flights of stairs per week*, *or walk for thirty minutes daily*, *or some kind of programme that somebody can just take away home from the hospital and think*, *"Right*, *I’m beginning this*, *I’ve got to do week one*, *week two*, *week three*, *week four"*, *and then even if they were just a call at the end of each week*, *just from somebody at the hospital*, *just to sort of hold them to account” (Partcipant-23*, *F*, *lines 84–89)*.

For others the hospital and home environments contributed to the isolation often associated with CF and therefore stated a preference for engaging in PA within the wider community. The wider community may also offer a greater range of activities than those available with the confines of a hospital or home-based programme.

“*I think CF can be quite isolating*, *where you’re kind of forced to stay indoors more than you’d like to*, *so I think having somewhere that’s not a hospital and then not your home*, *but somewhere else that you can go to [to engage in PA]” (Partcipant-26*, *lines 371–373)*.

The use of technology, in particular online video calling was suggested as a potential method to overcome the isolation resulting from segregation in CF. This may allow participants to benefits from the social aspects of engaging in PA as a group as well as provide the opportunity for peer support. Participants and clinicians acknowledged the potential benefits of such an intervention:

“*online sounds quite a good idea*, *so everyone could train*, *everyone with the same condition can do activity*, *and then I reckon in the hospital would be good as well*.*” (Partcipant-03*, *lines 272–273)*.

“*Could you do this as in tele-medicine*? *You know*, *a group of ten patients*, *it could be in North Wales or wherever*, *and if they all have exercise bikes set up*.*” (Clinician-03*, *lines 472–473)*.

#### 6.4.3. Intervention design–When should PA be promoted?. *(You’ve got giraffes on the wall*. *(Partcipant-02*, *line 626))*

As identified during phase 1 the transition from paediatric to adult care is an important period in the life of an individual with CF and often coincides with a worsening of disease severity, increased independence and responsibility for treatment as well other significant events such as moving away from home, employment or further study. The ‘giraffes on the wall’ are symbolic of the nurturing and friendly environment of a paediatric clinic, following transition to adult care there are no more giraffes on the wall. Participants (clinicians and individuals with CF) reported that PA should be encouraged from an early age;

“*Early*. *As early as possible” (Partcipant-04*, *line 167)*.

“*Oh*, *from day one” (Clinician-02*, *lines 438)*.

With an increase in PA promotion and support throughout the transition period;

“*I think maybe to definitely start*, *always try and start as early as possible*, *definitely*, *but take particular care and attention when they’re teenage” (Partcipant-21*, *lines 88–89)*.

“*Teenage*, *yes*, *because you kind of go off a little bit*. *Well*, *I did a little bit then…So that probably would be a really good time*, *because then you could do quite a lot of damage in those years if you’re not doing anything…” (Partcipant-23*, *lines 210–217)*.

“*…when they’re active as children and have got active families*, *when they drop off as a teenager*, *because all teenagers are naughty*, *they’re easier to regain it later on*, *but if they’ve never been active and they don’t have an active family*, *it’s never been something they do*, *it’s very hard for them to start with [lung function] of 40 to 50% as mid-twenties” (Clinician-02*, *lines 273–277)*.

#### 6.4.4. Intervention design–What action is required?. *(It’s more of a long game*, *isn’t it*?*” (Partcipant-21*, *lines 473–478))*

Exploration of how such support may be offered and how an intervention could be structured to meet the previously discussed objectives provided valuable information for the intervention design. A number of factors were previously discussed in phase 1, these included providing a variety of activities and opportunities for PA, the use of self-monitoring as a motivational tool and education for individuals with CF and parents. Engaging families in PA promotion was also constructed as an additional factor to consider when designing an intervention. It was reported that the family could have a role in holding individuals with CF to account with regards to adhering to a PA programme as well as making PA more enjoyable. For example:

“*…if you just say*, *"Oh*, *there’s an intervention"*, *they won’t be motivated*, *but then if you say there’ll be support and the family will be involved*, *I think they’re more likely to do it*.*” (Partcipant-03*, *F*, *lines 566–567)*.

Physical symptoms and uncontrolled set-backs were identified as barriers to PA during phase 1. Participants conceded that there was little that they could do to control for or plan for these. It was perceived that as part of an intervention it would be important to acknowledge that there may be set-backs and to be prepared to adapt to this to limit their impact, particularly in terms of the psychological impact such as causing frustration and becoming demoralising. For example:

“*…in terms of when uncontrolled set-backs and things were really really hard for me to deal with*, *not even necessarily from like a physical point of view*, *but just it’s so defeating when you get*, *especially if you*… *Because you used to set yourself milestones*, *"I want to be able to do this*, *this week"*, *and invariably I would set things that were a little bit unreasonable*, *and it would take me longer than I thought*, *but it felt really good when I got there*, *and then if that gets taken away from you by a chest infection*, *it’s really*, *really tough to then go back” (Partcipant-09*, *lines 184–188)*.

From a clinician perspective, the approach to PA promotion among adults with CF is the same as for that of the non-CF population. The challenge faced in a CF population is that the consequences of inactivity are greater than in non-CF peers. For example:

“*I don’t think there’s anything clever about CF exercise compared to exercise in the general public*. *It’s exactly the same…the consequences are greater*, *but the mechanisms and the strategies to get them to do exercise should be exactly the same…” (Clinician-1*, *lines 449–453)*.

From the outset of the research, the term ‘intervention to promote physical activity among adults with CF’ has been used to describe the end point of the formative action research process. There has been no definition of what an intervention is or any parameters to work within when designing the intervention. It was anticipated that the resulting intervention may mirror examples of exercise interventions within the literature, with the addition of specific objectives to target behaviours identified in earlier phases. These exercise interventions are typically structured programmes with or without supervision delivered in home-, community- or hospital-based settings. Typically, such interventions are delivered over a discrete period ranging from ~8 weeks to ~3 months. However, the key message resulting from the current research is that the promotion of PA in this population would be most effective as part of routine CF care rather than as a bespoke intervention. Participants suggested that PA promotion should form an integral part of their care and be given the same emphasis as other treatments. This would include regular contact with professionals reinforcing PA. For example:

“*So for me*, *I think it’d be better if it [PA] was a bigger topic in clinic in general*, *so yes*, *I would move away from this intervention whatever*, *more to just make it a more rigorous part of the treatment regime in the first place” (Partcipant-30*, *lines 245–248)*.

“*I don’t think there’s going to be any one kind of thing*. *I think it’s got to be all of it linking together*. *I’ve got no doubts about that… I think you’ve got to address all the barriers at once” (Partcipant-04*, *lines 467–470)*.

Participants emphasised the importance of the relationship between healthcare professional and individual with CF, suggesting than an individualised approach, developed through building a rapport and understanding individual needs is essential to increasing PA. For example:

“*…you need to find out what matters to them…what does matter to you*? *Does it matter to you that you’re physically capable to climb the stairs in your house*? *Does it matter to you that you’re physically capable to keep up with your peers*? *If it matters to you*, *shall we do something about it*? *And it’s that approach*, *rather than*, *"Are you booking us in today*?*" (Partcipant-04*, *lines 308–315)*.

One participant highlighted the unique nature of the relationship between individuals with CF and the CF team, describing it as follows:

“*It’s different with CF*. *You’re with the team*, *aren’t you*, *for a long time*?…*Even when you’re a kid*, *you’re with them for a long time*, *then you move to adult*, *and then you’ve got to*, *hopefully*, *be with them for a long time…but you are going to build up a relationship*, *aren’t you*, *with these people*? *It’s more of a long game*, *isn’t it*?*” (Partcipant-21*, *lines 473–478)*.

This quote encapsulates one of the unique aspects of PA promotion in this population, in that there is not a pre-determined end date or discharge representing the natural conclusion of an intervention.

### 6.5. Resources and policy

Phase 5 of the P-P model outlines the assessment of the budgetary, staffing and resources available to support an intervention as well as identification potential barriers and facilitators of an intervention at an administrative and policy level [17]. This phase was primarily conducted during the clinician focus group meeting.

#### 6.5.1. Assessment of resources

The promotion of PA is currently recommended as part of routine CF care, with specific recommendations for best practice outlined for physiotherapists [4]. In addition to this, numerous sources of information and resources useful in the promotion of PA were identified, including written information, mobile applications and online video content from existing organisations. Although there is no requirement to employ staff with a background in exercise science or an existing pathway for such individuals to work in CF the service participating in the current research employed two full-time members of staff to deliver exercise services. There was also access to a small facility to conduct exercise testing and prescription.

“*it’s [exercise provision] just this is an unmet need*, *and to a certain extent*, *it’s unrecognised*.*” (Clinician-01*, *lines 610–611)*.

### 6.6. Barriers to implementation

Reported barriers to implementation included additional staffing requirements, a lack of space, an underrepresentation in clinic and an inability to perform CPETs. For example:

“*We don’t have the staff and we don’t have the place to bring them” (Clinician-02*, *lines 604–605)*.

“*The only thing we’re missing is the gold standard cardio-pulmonary exercise testing on all the patients… but you’re talking money there” (Clinician-02*, *lines 618–620)*.

“*there’s no doubt now that if we didn’t have an exercise physiology team*, *we wouldn’t be getting one*. *Now that’s not because they’re not valuable*, *it’s just because this organisation’s really strapped for cash…and so what we’ve got is what we have*, *and we’re very grateful and it’s good” (Clinician-01*, *lines 634–638)*.

### 6.7. Facilitators for implementation

There are specialist CF centres established throughout the UK with specialised teams already in place to support individuals with CF, adding to current services could provide the opportunity to promote PA whilst maintaining familiarity. Although employing an appropriately trained and experienced member of staff has financial implications. For example:

*“You’d need another Band 6 [exercise professional], somebody with some experience and a real interest in it [CF and exercise], so that salary straight away per year’s a lot…”(Clinician-02, lines 595–596)*.

The primary objective of CF care is often to slow the rate of decline and so the aim of an intervention may not be to produce drastic short-term improvements but rather to have improved outcome long-term. As illustrated here:

*“It’s maintaining the levels. Reaching an optimum level for that patient and then maintaining it, because it’s a slippery slope with CF.” (Clinician-01, lines 503–504)*.

As referred to as part of the assessment of resources there are a range of resources available to support the promotion of PA in individuals with CF. The role of the clinical team is to be aware of these resources and to be able to signpost patients to relevant resources and services at an individual level.

## 7. Phase 2 discussion

The aim of the current study was to understand the ecological correlates of PA in adults with CF and to use these findings to inform the development of an ecological approach to PA promotion in this population. In doing so a formative approach was employed, involving individuals with CF, their families (where applicable) and clinicians.

Principle predisposing factors which represent suitable targets for increasing PA in adults with CF relate to removing the barriers associated with disease progression, increased symptom burden and uncontrolled set-backs. A key facilitator of PA for the participants with CF was improved wellbeing, which is typically associated with improvements in clinical measures including lung function and fitness. Both participants with CF and their clinicians reported enjoyment as a significant facilitator of PA, although this represents a challenge for PA promotion as participants describe PA being enjoyable as a child when the impact of CF is less pronounced and enjoyment turning to necessity and treatment with disease progression. With participants describing their PA as ‘normal’ during childhood. Participants with CF reported that the impact of CF becomes more pronounced during adolescence, which contributes to the shift from enjoying PA to PA being viewed as a treatment. This period is also associated with transition from paediatric to adult care. A systematic review of qualitative studies exploring correlates of PA reported enjoyment of PA a motivation for PA across all age groups [39]. In older adults participation in prescribed PA was also maintained through enjoyment [39]. Enjoyment is clearly important, however enjoyment of PA is highly variable between individuals, as such PA promotion should adopt an individualised approach, encourage individuals to engage in a variety of activities to promote enjoyment, which may improve self-efficacy and motivation for PA [29].

Enabling factors which represent targets of PA promotion include providing a range of activities to encourage enjoyment of PA. Additionally, education and specific direction to help to overcome some of the challenges of being active with CF, particularly managing symptoms and set-backs. Participants reported that the environment did not present barriers to PA. In terms of intervention delivery there appears to be scope to promote PA in a number of settings including at hospital, home, community and online, although this requires an awareness of local resources and individual needs.

From the perspective of participants with CF and their families the clinical team and in particular the presence of an exercise professional appears to be central to reinforcing PA behaviour. Regular contact at each clinic visit with such an individual and the development of a rapport appears to be key facilitator of PA. Whilst neither the participants with CF nor their clinicians alluded to how this rapport may be established this finding is consistent with previous research in this population. Participants with CF participating in a counselling intervention reported that they perceived consistent contact with a health care professional beneficial for their psychological health and valued informal long-term enduring relationships with health care professionals [40]. In addition to this, the role of the family in supporting individuals with CF was reported as a reinforcing factor, important to the promotion of PA. Participants reported that their family played a role in holding them accountable for the PA levels as well as making engaging in PA more enjoyable.

Participants with CF and their clinicians suggested that the overall aim of an intervention to promote PA should be to improve the overall ‘wellbeing’ of individuals with CF. This encompassed improvements in key outcome measures, increased PA and an ability to engage individuals with CF in PA. There was a belief among participants with CF that PA should have the same emphasis as other aspects of the management of CF. Both participants with CF and their clinicians highlighted promoting PA as early as possible as an important factor in increasing PA in individuals with CF, with additional support given during the transition from adolescent to adulthood.

The key message resulting from the current research is that the promotion of PA in this population would be most effective as part of routine CF care rather than as a bespoke intervention. To achieve the aims of improving wellbeing and physical function in individuals with CF, as well as engaging them in a programme of PA there appears to be a requirement to have a dedicated healthcare professional within the CF team. An individual with expertise in PA who understand CF and has the capacity to provide individually tailored advice through frequent contact and support. Although PA is recommended as part of routine CF care there no requirement for CF services to include an exercise professional and no standardised role for an exercise professional within CF MDTs. A number of UK centres are now employing individuals to oversee exercise provision but these roles remain largely undefined [41,42]. The model of PA promotion suggested in the current study is considerably different to the conventional exercise-training model that has constituted the majority of research in this area. Existing exercise training interventions do not consider local environments and interests at an individual level, are resource intensive and often lack long-term sustainability [5]. The integration of exercise professional led PA promotion into CF care may represent a more sustainable and effective model to increase PA in individuals with CF. In addition to the role of an exercise professional participants with CF also alluded to the inclusion of a psychologist in supporting the promotion of PA. Clinical psychologist are already present within CF MDTs and play an active role in supporting patients, however the nature of the role described here may be more akin to the role of a Health Psychologist. Health psychology is concerned with the psychological, behaviour and social factors contributing to health and typically takes a person centred approach to behaviour change and health promotion [43], whereas clinical psychology primarily focuses on treating psychological disorders. Whilst the CF clinic was identified as the primary location for the promotion of PA to take place participants also expressed a desire to have multiple options including access to community resources, home-based programmes and thorough the use of online technology and media. The use of technology in promoting PA in individuals in CF is an emerging area of research and represents a feasible and acceptable method of intervention delivery [44], the importance of this area of research has been highlighted throughout the COVID-19 pandemic, during which time the delivery of face-to-face services was limited. Clinicians suggested that the promotion of PA in adults with CF is similar to in the general population, indeed a number of the correlates of PA discussed in Phase 1 were comparable to non-CF populations. Aspects unique to individuals with CF related to the additional consequences of inactivity for health, the physical and psychological barriers associated with the condition and the long-term nature of the relationship between individuals with CF and their clinicians. It is there important for clinicians working with individuals with CF to acknowledge that there may be set-backs and to be prepared to adapt to this to limit their impact, particularly in terms of the psychological impact. Supporting individuals with CF with setting goals, managing set-backs and overcoming such psychological barriers could form part of the role of exercise physiologists and psychologists who were reported as key individuals in delivering an intervention. The long-term enduring relationships between health care professionals and individuals with CF provides a unique opportunity to support patients, more broadly and in terms of PA promotion. Previous research has demonstrated that individuals with CF value this long-term presence of a health care professional [40]. However, self-regulation is consistently reported as a correlate of PA, as such health care professionals face the challenge of providing support whilst also promoting self-regulation and avoiding participants reliance on their support [31].

There are numerous resources available to support clinicians and individuals with CF to increase PA, although clinic space, equipment and staffing represent barriers to implementation. Access to resources is commonly reported as a limitation to adherence with PA recommendation across UK CF services, despite PA being valued among clinicians [45].

New drugs targeting the basic defect of CF have provided hope for individuals with CF however, with these advances comes new opportunities and challenges [46]. Such therapies offer the hope of reduced symptom and treatment burden and increase life expectancy which may provide increased opportunities for PA and alter perceptions and experiences of PA. Preliminary research has already begun to highlight potential for reducing discomfort associated with exercise and enhancing endurance among individuals receiving Orkambi [47]. It is therefore essential that researchers and healthcare professionals continue to engage with individuals with CF to understand PA behaviour in the era of modulator therapies.

### 7.1. Strengths and limitations

There are a number of strengths apparent in the present study. Firstly, the formative research design allowed detailed exploration of factors influencing PA and discussion with individuals with CF, their families and clinicians providing novel insight into the correlates of PA. Furthermore, the research advances the use of qualitative methodologies including participatory research and the use of pen profiles in this population. The findings from this study also have important implications for the design of future interventions promoting PA in adults with CF. Participation was voluntary, as such, the self-selecting nature may have resulted in a sample of patients already motivated to be physically active. Additionally, there was a relatively small sample of participants from a single centre. Although the transition from paediatric to adult care was discussed, the participants did not all attend the same paediatric centre and those that did attend the same paediatric centre, may have experienced a different process due to differences in the age of participants and changes in over time. However, the study design and analysis methods allowed for the collection of in-depth qualitative data from individuals with CF, families and clinicians perspectives. Given the constructivist approach underpinning the qualitative methods the generalisability of the results was not based on conventional statistical probability (which would rely on a larger sample size) but was based on the detailed exploration of multiple perspectives from a diverse sample of individuals with CF of varied age, disease severity and experiences [48]. The methods used are not intended to provide estimates of physical activity levels but rather seek to better understand physical activity behaviour in this population. Participants did not have access to new triple combination modulator therapies during the study as they were not yet widely available, it is therefore not possible to assess the potential impact of these drugs on perceptions and experiences of PA.

## 8. Conclusion

The promotion of PA in adults with CF may not be best achieved through the delivery of a single intervention but through the role of an exercise professional as part of routine CF care long-term. PA promotion should begin during paediatric care and be reinforced throughout an individual’s life with additional support during adolescence. The role of an exercise professional should be to identify the principle predisposing, enabling, reinforcing factors influencing PA behaviour at an individual level in order to remove barriers to PA, engage patients and improve ‘wellbeing’.

## 9. Recommendations

PA promotion should form part of routine clinical care, with designated exercise professionals available to identify barrier and facilitators to PA, reinforce PA behaviour and support patients at an individual level. PA promotion should involve family members from an early age and throughout the course of an individuals’ life, although support should be intensified during adolescence. The role of an exercise professional as part of the CF MDT is not currently a requirement of CF services, with PA promotion typically a responsibility of the CF physiotherapist. In order to establish the role of an exercise professional as part of the CF MDT an accreditation pathway and standardised role are required.

## Supporting information

S1 File Individual patient interview schedule. (PDF)

S2 File Patient focus group schedule. (PDF)

S3 File Clinician focus group schedule. (PDF)
