# Supplementary material for: Developing an ecological approach to physical activity promotion in adults with Cystic fibrosis
Source: PLoS One. 2022 Aug 1;17(8):e0272355. doi: 10.1371/journal.pone.0272355 (PMC9342769; doi:10.1371/journal.pone.0272355)
Supplement: S1 File — (PDF) [file pone.0272355.s001.pdf]

Individual participant interview schedule

Date: DD/MM/YYYY **Location:** **Participant ID: CFCS … …**

Introduction

Hi, my name is [*researchers' name*]. I'm a researcher at Liverpool John Moores University and I am currently working with the Cystic Fibrosis (CF) team at Liverpool Heart and Chest Hospital to try and understand more about your experiences of living with CF and your thoughts and opinions about physical activity more generally. I'm here to get **your** perspective on these things as an individual with CF. There's no right or wrong answers.

Please try and give as much information as possible, using your own words and give examples where you would like to offer these. The interview will take around 45-60 minutes, which will include 13 questions relating to your general health and physical activity. I'd also like you to know that this interview will be audio recorded and transcribed – which means writing out our conversation like a script, so I can refer back to it. Additionally, I may make some notes as we are talking so that I can refer back to some of the points discussed. The things you say will remain confidential and your name or other details which may be used to identify you will not be reported. This research has been reviewed and given approval by an independent group of people known as a research ethics committee *[insert REC and reference number]*.

Do you have any questions before we start?

Theme	Main Question	Sub-question(s)	Prompts/probes
**[Transition statement]** I'd like to start by finding out a little bit more about you.			
**General health**	1. To begin with could you tell me a bit about yourself?		
**General health**	2. Can you tell me about your experiences of living with CF?	How do you think you can best manage your health?	Thoughts/feelings<br>Impact on self/ family
**[Transition statement]** It's really interesting to learn more about you and your health. I'd now like to find out a little bit more about your thoughts about physical activity.			
**Physical activity**			
General	3. What does the term physical activity mean to you?		What does the term physical mean to you?<br>What does the term activity mean to you?
[Provide definition] - Physical activity is defined as any activity that increases energy expenditure and may include sport, work, household activities and transport such as walking or cycling.			
	4. Can you describe what your current physical activity behaviour is like?		Types of PA
	5. Can you describe any challenges to being as active as you'd like?		Thoughts/feelings
**[Transition statement]** Thank you, I'd now like to focus on factors that may lead you to be more or less active.			
Predisposing	6. What are your personal attitudes towards having a physically active lifestyle?<br>Is it or why is it important to be physically active?	Do you enjoy being physically active?	Thoughts /feelings

	7. What would you like to do to be more physically active?		Motivations to engage with PA<br>Goals relating to PA<br>Daily lifestyle activity
Reinforcing	8. Who, if anyone, do you engage in physical activity with?	Can you describe any physical activities you do as a family?<br><br>How do they influence you?	Types of PA<br><br>Positively/negatively
	9. How, if at all, do you feel that the CF team influence your physical activity? In what ways do they influence your physical activity?		Positively/negatively
Enabling	10. How would you describe your past experiences with physical activity to someone else?	How has PA changed for you over the years?	
	11. How if at all, does your neighbourhood (the area you live and your local community) influence your physical activity? Does it make being active easy? Does it make being active hard?	Does it make being active easy?<br>Does it make being active hard?	
	12. Please could you tell me how you feel about your ability to take part in physical activity?		*[reference to activities discussed in Q4]*<br>Provision of information about CF and physical activity<br>Satisfaction with information
	13. Finally, one of the reasons for my research is to understand how to increase physical activity amongst individuals with CF. So, do you have any ideas about what would be helpful to support physical activity engagement in individuals with CF?		Types of support<br>Anything that would/wouldn't work<br>Why?<br>How could it work?<br>What would it look like

**Prompts/Probes are there to be used as a guide for the interviewer. They are key words/phases to help the researcher ask questions and elicit responses from the participant. Prompting questions will be used in a conversational manner and only when deemed appropriate.*

Transition – It has been really useful finding out more about you. Let me briefly summarise the information we have discussed.

Closing

[Provide a summary of discussion]. I appreciate the time you took for this interview. Is there anything else you think would be helpful for me to know?

I should have all the information I need, thanks again.
