# Supplementary material for: Developing an ecological approach to physical activity promotion in adults with Cystic fibrosis
Source: PLoS One. 2022 Aug 1;17(8):e0272355. doi: 10.1371/journal.pone.0272355 (PMC9342769; doi:10.1371/journal.pone.0272355)
Supplement: S2 File — (PDF) [file pone.0272355.s002.pdf]

Patient focus group schedule

Date: DD/MM/YYYY

Location:

Participants:

Hi, my name is [*researchers' name*]. I'm a researcher at Liverpool John Moores University and I am currently working with the Cystic Fibrosis (CF) team at Liverpool Heart and Chest Hospital to involve patients with CF, their families and clinicians in a process to develop an intervention to promote physical activity in adults with CF. The reason we're here today is to discuss the perceived barriers, facilitators and opportunities for physical activity participation and how this information can inform the development and delivery of a PA intervention for individuals with Cystic Fibrosis.

This research has been reviewed and given approval by an independent group of people known as a research ethics committee *[REC and reference number]*. I'd also like you to know that this meeting will be audio recorded and transcribed – which means writing out our conversation like a script. The recording and transcript will allow me to revisit our discussion for further analysis. Additionally, I may make some notes as we are talking so that I can refer back to some of the points discussed. The identities of all participants will not be included in this transcript or any other reports.

The goal of a focus group is to stimulate discussion to find out opinions and attitudes about a topic of interest, in this case physical activity in patients with CF. To allow the focus group to cover the pre-determined topics and flow smoothly, I'd like to go over some principles of focus groups.

1. This is a confidential discussion in that I will not report your name or what you have said. I would also like to request that this discussion remains in this room and is not discussed outside of the focus group.
2. Names of participants in any of the interviews will not even be included in the final report about this meeting.
3. I would like to stress confidentiality as I would like an open discussion. I want you to feel free to comment without concern that your comments will be repeated later and possibly taken out of context.
4. There are no "wrong answers", just different opinions and views.
5. Let me know if you need a break.

The focus group will take around 45-60 minutes, which will include questions in four separate phases.

Does anyone have any questions before we start?

Diagnostic phase	Objective	Themes	Questions
**[Transition statement]** I'd like to start by asking some questions to try and understand more about individuals with CF and present some of the themes (determinants of PA) identified during the phase 1 interviews.			
		Stage 1 will be informed by responses given during individual patient interviews and the themes subsequently identified.	
**Stage 1:** Social diagnosis	Ask and answer key questions related to the health issue	What is the health problem? (Associated with inactivity in CF?)<br><br>Who is the priority population? (How is this defined, what characteristics do they share?)<br><br>What are the important behaviours for inactivity? How do these differ between groups?<br><br>Describing the context of an intervention (population, setting and community).	How if at all, does CF impact quality of life in patients with CF?<br><br>How if at all, does CF impact physical activity in patients with CF?<br><br>Who is the priority population?<br><br>How is this population defined and what characteristics do the share?<br><br>Who do you think would be responsible for delivering an intervention designed to increase physical activity in patients with CF?

<table>
<tr><td colspan="4">[Transition statement] Thank you for sharing your thoughts with me. I’d now like to move on to some questions relating to creating measurable behavioural outcomes for a potential intervention.
[Present logic model devised based on findings from phase 1 – displaying what change is needed to prevent, manage, or reduce identified health problems associated with inactivity. Outlining the proposed mechanisms of change, the determinants expected to influence and the behavioural and environmental outcomes that will address the health problem]</td></tr>
<tr><td rowspan="2">Stage 2:
Epidemiological diagnosis</td><td rowspan="2">Create measurable, time-limited, health-related objectives. The success of the program will ultimately be judged by these objectives</td><td colspan="2">Clinical data and cross-sectional PA data may also be used to inform this stage.</td></tr>
<tr><td>State expected outcomes for behaviour and environment

Specify performance objectives for behavioural and environmental outcomes.

Select determinants for behavioural and environmental outcomes.

Determine the aims of an intervention designed to increase physical activity in patients with CF?</td><td>What is the priority health problem associated with inactivity?

Which factors are associated with these behaviours?

Which methods do you believe may be effective in changing these behaviours? (as highlighted in logic model)

What needs to happen to enable these changes to take place? Prompts/probes (E.g. training, resources, communication).

Is it possible to measure this? If so, how could this be measured?</td></tr>
<tr><td colspan="4">[Transition statement] Thank you for sharing your ideas and developing these objectives. I would now like to discuss some of the smaller sub-objects which may be used to ensure that the main objectives are met, in doing so I would like you to consider which are related to behavioural factors and which are related to environmental factors.</td></tr>
<tr><td rowspan="2">Stage 3:
Behavioural and environmental diagnosis</td><td rowspan="2">Identify key environmental and behavioural factors; these will become sub-objectives that direct planning for intervention activities</td><td colspan="2">This phase will be informed by findings in stage 2 and any accompanying behaviour change theories/techniques adopted.</td></tr>
<tr><td>[Define] Environment - Interpersonal environment (family, friends, clinicians etc.), Organisation environment (Clinic, hospital, CF trust), Community environment (geographic, social environment, CF community)

Behaviour (beliefs, self-efficacy, perceived norms)</td><td>What are the priority behavioural and environmental contributors to inactivity in CF?

Who if anyone, can influence environmental conditions?

Which determinants of PA are important in achieving the [objectives outlined] and how might these be modified? (consider for each separate objective)</td></tr>
<tr><td colspan="4">[Transition statement] I’d now like to consider how it may be best to meet these objectives and how they may relate to the principle predisposing, reinforcing and enabling factors we discussed at the start.</td></tr>
<tr><td>Stage 4:
Educational</td><td></td><td colspan="2">Identify, sort, and categorise the predisposing, reinforcing, and enabling factors that influence health behaviours identified in phase 1.</td></tr>
</table>

and ecological diagnosis	Develop a unique plan to achieve each sub-objective from step 3; Consider predisposing, reinforcing, and enabling factors, and use theory	Predisposing (motivation, opportunities, lifestyle)<br><br>Reinforcing (Peers, family, health, enjoyment)<br><br>Enabling (Cost, location, facilities, transport)	The key predisposing factors identified during phase 1 were […]. How might the objective outlined above be met considering these factors?<br><br>The key predisposing factors identified during phase 1 were […] How might the objective outlined above be met considering these factors?<br><br>The key predisposing factors identified during phase 1 were […] How might the objective outlined above be met considering these factors?
**[Transition statement]** Finally, I would like to discuss the feasibility of delivering such a plan.			
**Stage 5:** Administrative and policy assessment	Assess capacity and resources available to implement programs and change policies such that step 4 sub-objectives can be met	Budgetary and staff requirements and availability, barriers/limitations to overcome, and available policies to change or support.<br><br>Health education<br><br>Policy, regulation, and organizational structures	What are the existing policies and practices that could be leveraged to support the intervention?<br><br>What are the existing organisation/groups that could help to support the intervention?<br><br>What aspects of the natural or built environment could be harnessed to support the intervention?
**Close**<br><br>*[Provide a summary of discussion].* I appreciate the time you took for this focus group. Is there anything else you think would be helpful for me to know?<br><br>I should have all the information I need, thank you.			

Findings will inform the PROCEED component of the PRECEDE-PROCEED
